# Disseminated Neonatal Herpes Caused by Herpes Simplex Virus Types 1 and 2

**DOI:** 10.3201/eid1302.060907

**Published:** 2007-02

**Authors:** Aleksandra Knezevic, Jelena Martic, Maja Stanojevic, Sasa Jankovic, Jasminka Nedeljkovic, Ljubica Nikolic, Srdjan Pasic, Borisav Jankovic, Tanja Jovanovic

**Affiliations:** * University of Belgrade, Belgrade, Serbia; † Mother and Child Health Institute Dr Vukan Cupic, Belgrade, Serbia; ‡ Institute of Immunology and Virology Torlak, Belgrade, Serbia

**Keywords:** Neonatal, herpes, disseminated, infection, herpes simplex virus, dual infection, dispatch

## Abstract

Disseminated neonatal herpes simplex virus (HSV) infection is characterized by progressive multiple organ failure and high mortality rates. It can result from infection with either HSV-1 or HSV-2. We report a case of disseminated neonatal herpes that was caused by HSV-1 and HSV-2.

Neonatal herpes simplex virus (HSV) infection is among the most severe perinatal infections. Most (85%) neonatal HSV infections are acquired during delivery, although in utero (5%) and postnatal (10%) infections do occur (*1*). The risk for transmission to the newborn is much higher in women with primary HSV infections (*2*). Neonatal herpes can be localized to skin, eyes, and mouth (≈45% of cases), involve the central nervous system (≈30% of cases), or can cause disseminated infection involving multiple organs such as liver, lungs, adrenal glands, and brain (≈25% of cases).

Disseminated infection is the most severe form of neonatal herpes, with a mortality rate of 85% for untreated neonates (*3*). It is usually observed when the infant is 5–9 days old; signs include irritability, seizures, respiratory distress, jaundice, bleeding diatheses, shock, and often vesicular exanthema (*3**,**4*). Early treatment with high-dose acyclovir reduces the mortality rate (*5*). Early recognition of disseminated infection is difficult because of nonspecific symptoms and signs of sepsis and because initiation of antiviral therapy is often delayed (*1*). The high risk for death requires prompt diagnostic evaluation that includes testing by HSV DNA PCR as the preferred method or virus culture (*6*, *7*).

Neonatal herpes can result from infection with either HSV-1 or HSV-2; the latter is associated with a poorer prognosis (*7*). We report a case of disseminated neonatal herpes infection with HSV-1 and HSV-2.

## The Case

A full-term infant girl had febrile illness and lethargy and fed poorly at 3 days of age after a normal vaginal delivery with prolonged expulsion of placental membranes. Initial laboratory investigations showed a normal leukocyte count (11,100 cells/μL), a slightly elevated C-reactive protein level (18 mg/L), and elevated levels of liver enzymes (aspartate aminotransferase [AST] 283 U/L and alanine aminotransferase [ALT] 111 U/L). A screen for sepsis was performed and she was treated with broad-spectrum antimicrobial drugs. On day 4 of life, signs of respiratory distress appeared and intermittent mechanical ventilation was initiated. A chest radiograph showed streaky and patchy bilateral pulmonary opacities and right-side pleural effusion. Neurologic examination showed mild hypotonia. On day 6 of life, she was still febrile with thrombocytopenia (56,000 cells/μL), an increased C-reactive protein level (32 mg/L), and signs of fulminate liver failure (AST 13,740 U/L and ALT 3,180 U/L) and marked coagulopathy. Despite full intensive care support, she died of rapidly progressive multiple organ failure on day 9.

Postmortem findings showed widespread necrosis of lungs, liver, and adrenal glands. Serologic investigations showed no immunoglobulin M (IgM) and IgG antibodies for HSV-1 and HSV-2. An ELISA (Virion/Serion, Würzburg, Germany) detected IgG antibodies for rubella virus, cytomegalovirus, varicella zoster virus, parainfluenza virus, adenovirus, and coxsackie B virus; however, these results were not indicative of active infection. Blood and urine bacterial cultures were negative. Retrospective virologic examination of postmortem specimens (tracheal aspirate, liver, lungs, and stomach) in different cell lines (Vero, RD, L20B) showed cytopathogenic virus, which was suggestive of HSV that was identified by PCR.

Viral DNA was extracted from all postmortem specimens (tracheal aspirate, liver, lungs, and gut) and all virus-positive cultures of tracheal aspirate, liver, lungs, and stomach in different cell cultures (Vero, RD, L20B) by using the QIAamp DNA Mini Kit (QIAGEN, Valencia, CA, USA). The DNA was then used for HSV DNA PCR. Target DNA was amplified with primers for the HSV-1 thymidine kinase gene (Fw 5′-AGCGTCTTGTCATTGGCGAA-3′ and Rev 5′-TTTTCTGCTCCAGGCGGACT-3′) and for the HSV-2 DNA polymerase gene (Fw 5′-CGTCCTGGAGTTTGACAGCG-3′ and Rev 5′-CAGCAGCGAGTCCTGCACACAA-3′) (*8*). A 342-bp band for HSV-1 and a 445-bp band for HSV-2 were found in all postmortem specimens (Figure) and in all virus-positive cultures examined.

**Figure:**
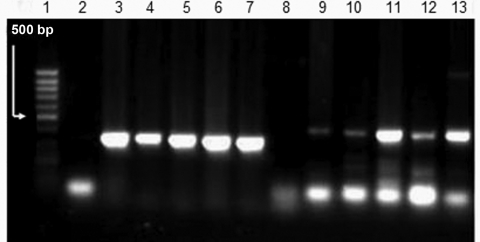
Results of herpes simplex virus (HSV) type 1 and type 2 PCRs in postmortem specimens. Lane 1, 100-bp DNA ladder; lane 2, negative control HSV-1; lane 3, positive control HSV-1; lane 4, tracheal aspirate HSV-1; lane 5, liver HSV-1; lane 6, lung HSV-1; lane 7, stomach HSV-1; lane 8, negative control HSV-2; lane 9, positive control HSV-2; lane 10, tracheal aspirate HSV-2; lane 11, liver HSV-2; lane 12, lung HSV-2; lane 13, stomach HSV-2.

Nucleotide sequence analysis was performed by using the ABI Prism BigDye 3.1 sequencing system (Applied Biosystems, Foster City, CA, USA) and showed identical sequences in different specimens. When these sequences were compared with those available in the GenBank database by using the BLAST tool (www.ncbi.nlm.nih.gov/BLAST/), the highest similarity was observed for relevant HSV genes, namely HSV-1 strain CL 101 and HSV-2 strain KN 53690.

Retrospective serologic examination of maternal samples and avidity tests at 3 different time points detected IgM and IgG antibodies initially for HSV-1 and subsequently for HSV-2 (Table); the increase in IgG avidity to both virus types correlated with primary infection.

**Table:** Maternal serologic status for herpes simplex virus (HSV)–1 and HSV-2 at different time points after delivery*

Time after delivery, mo	HSV-1†		HSV-2†	
	IgM (IU/mL)	IgG (IU/mL)	IgM (IU/mL)	IgG (IU/mL)
2	170	836 Avidity 57%	35	–
6	145	1,019 IU/ml Avidity 90%	50	135 Avidity 50%
9	57	550 IU/mL	–-	55 Avidity 77%

*IgM, immunoglobulin M. †Reference values: IgM positive >30 IU/mL; IgG positive >30 IU/mL.

## Conclusions

Neonatal disseminated HSV infection is most frequently caused by HSV-2, although HSV-1 can also be the cause. To the best of our knowledge, our patient is the first PCR-confirmed case of disseminated neonatal herpes caused by concomitant infection with HSV-1 and HSV-2.

Prompt diagnosis was difficult because of the early appearance of nonspecific symptoms (day 3), signs of respiratory distress (day 4), and rapid development of multiple organ failure (day 6). Oral and skin vesicular lesions were not detected, and the mother had no history of herpes infection. Serologic HSV status of the newborn was not of great clinical value. Postmortem virologic examination including viral isolation and HSV DNA PCR identified HSV-1 and HSV-2.

The results of retrospective serologic examination for maternal IgM and IgG antibodies to HSV-1 and HSV-2 and avidity tests suggested that primary maternal HSV infection occurred near the time of delivery. Because of the 2-month delay in obtaining maternal serologic results, whether the mother was infected by both HSV types concomitantly or successively near the time of delivery is unclear. These results suggest that the newborn acquired the infection during delivery, although in utero infection cannot be ruled out. The rapid onset of disseminated neonatal HSV infection (day 3) and development of multiple organ failure seen in this patient may be the result of concomitant infection with HSV-1 and HSV-2.
